# Integrating Dermocosmetics Into Acne Care in Latin America

**DOI:** 10.1111/jocd.70776

**Published:** 2026-03-01

**Authors:** Patricia Troielli, Jorge Moreno, Andrea Cortes, Paola Cardenas, Delphine Kerob, Adriana Gamarra, Brigitte Dreno

**Affiliations:** ^1^ School of Medicine, Department of Dermatology University of Buenos Aires Buenos Aires Argentina; ^2^ Dermavanz Salud (Private Practice), Centro Medico National del Noreste and Hospital Universitario Dr Jose E. Gonzalez Monterrey Mexico; ^3^ Dermatology Service Hospital Clinico Universidad de Chile Santiago Chile; ^4^ Dermatology Department National University Bogota Colombia; ^5^ La Roche‐Posay Laboratoire Dermatologique, L'Oreal Dermatological Beauty Division Levallois France; ^6^ Clinica Lima Derma Lima Peru; ^7^ Nantes Université INSERM, CNRS, Immunology and New Concepts in ImmunoTherapy, INCIT Nantes France

**Keywords:** acne vulgaris, cosmeceuticals, dermocosmetics, management, over‐the‐counter

## Abstract

**Background:**

Prescription acne products have proven efficacy and safety, yet management can pose a challenge. This review discusses the benefits of adding dermocosmetics to acne management.

**Methods:**

We add expert consensus with review of the literature to provide guidance for clinicians managing patients with acne in Latin America.

**Results:**

There is increasing evidence that dermocosmetics (over‐the‐counter cleansers, moisturizers, and sunscreens that contain acne‐targeting ingredients) can be a good alternative to prescription acne treatments as well as adjuncts. Milder forms of acne may be present in any age patient, but prepubertal acne and acne cosmetica may be particularly well suited to a dermocosmetic approach. More severe acne may need a dermocosmetic added if there is sensitive skin or poor tolerance to prescription medications, and when the patient or family does not wish to use antibiotics or other acne prescription treatments. Dermocosmetics may be used as adjuncts to any type of prescription therapy, but may be most effective when used with products associated with skin irritation such as topical retinoids or benzoyl peroxide. Appropriate dermocosmetics can also fortify the skin barrier and help to protect the skin microbiome.

**Conclusions:**

Acne management is complex and there can be adherence, tolerability, and efficacy problems. Dermocosmetics alone can be used in milder forms of acne or in maintenance post treatment, as a good compromise between efficacy and tolerability. As adjuncts, dermocosmetics can also decrease skin irritation and thereby increase adherence, can enhance the efficacy of prescription therapies, and can normalize dysbiosis in acne.

## 1 Methodology Used

This narrative review is based on an expert round‐table of six dermatologist co‐authors from the LATAM Acne Board on the role of dermocosmetics in acne, following a structured literature review in PubMed/MEDLINE (English and Spanish, studies from the last two decades) using key terms related to acne, dermocosmetics/cosmeceuticals, and Latin America, including clinical and observational studies while excluding non‐clinical or offtopic publications.

## 2 Introduction

Acne, a chronic inflammatory skin disorder, and its sequelae of post‐inflammatory hyperpigmentation (PIH) and scarring are both associated with a negative impact on sufferers' quality of life [1]. The pathophysiology of acne in Latin America is similar to that in other world regions; however, the natural history of acne in Latin American may differ due to demographics, racial or ethnic diversity, and exposome factors (climate, environment, diet) [1]. In addition, there is a heterogeneous population including a relatively high proportion of individuals with skin of color (Fitzpatrick skin phototypes III–VI) in Latin America, which can increase the challenges surrounding acne due to propensity to PIH and scarring [1, 2, 3, 4, 5].

In the opinion of the authors, acne is often a neglected disease likely due to low awareness about best strategies for management among both the lay public and healthcare professionals. Further, depending on the location within Latin America, there may be barriers to access of dermatologic care including, but not limited to, cost/availability of treatments, laxity/misuse or underuse of prescription drugs, and an online marketplace containing over‐the‐counter (OTC) products with poor effectiveness. While there is a paucity of studies of acne epidemiology from Latin America, a 2018 study in a public Brazilian secondary care center reported that acne was the fourth most common reason for consulting a dermatology, which is similar to data from other populations, particularly skin of color groups [6, 7, 8, 9]. Additionally, a 2021 report from Colombia showed a marked increase in adult female acne in a five‐year study involving almost 155 000 female patients diagnosed with acne [10].

**FIGURE 1:**
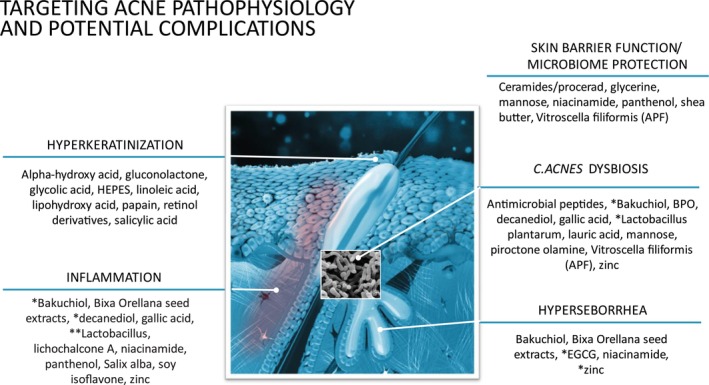
Anti‐acne ingredients categorized by type of action.*Additional/secondary action; **Fermented; APF = *Aqua Posae filiformis*; BPO = benzoyl peroxide; EGCG = epigallocatechin‐3‐gallate; PIH = post‐inflammatory hyperpigmentation.

There is increasing recognition that dermocosmetics (also referred to as cosmeceuticals) can play an important role in acne management [11]. These products are non‐prescription but contain ingredients that have benefits that go beyond that of the vehicle [11]. Recommendations for dermocosmetics should always include educating patients on appropriate sunscreens, cleansers, and moisturizers [11]. Both patients and healthcare professionals should be aware of the anti‐acne ingredients to look for (Figure 1) [11].

Although several of the studies and illustrative cases cited in this review involve specific branded dermocosmetic formulations, the recommendations proposed are based on ingredients, mechanisms of action and clinical profiles that can be extrapolated to dermocosmetic products with similar active ingredients and characteristics, rather than to any particular brand.

As noted above, the pathophysiology of acne is considered to be relatively similar across world regions; however, acne experts have increasingly become aware of the importance of optimizing skin barrier function with acne management [12]. Prakash et al. reported that the pH of facial skin in individuals with acne is higher than normal and contributes to stratum corneum instability [12]. Further, they speculate that skin barrier dysfunction may contribute to the waxing and waning natural history of acne and that use of skin barrier support measures may be integral in acne management [12]. Other researchers have shown that the skin microbiome is important, particularly the loss of balance between different *Cutibacterium acnes* phylotypes as well as interactions between *C acnes* and *Staphylococcus epidermidis* [13]. Both of these emerging aspects of acne pathophysiology support the rationale for use of dermocosmetics in acne management.

It has long been known that treatment adherence is poor in acne. A 2010 international study reported adherence rates as low as 37% with topical therapy in the Americas [14]. Further, cutaneous irritation and/or side effects have been reported to be major factors in poor adherence [15, 16]. Clearly, then, interventions that improve the tolerability of acne treatments—particularly in the beginning of treatment—can be encouraged. Despite the high incidence and burden of acne and its sequelae in Latin America, epidemiologic studies and interventional trials specifically conducted in this region remain limited. There is a clear need for more randomized controlled trials and real‐world studies that include patients with a wide range of phototypes, including the darker ones, and assess outcomes such as post‐inflammatory hyperpigmentation, scarring, and quality of life. Future research should also address structural barriers to evidence generation in Latin America, including heterogeneous healthcare systems, restricted access to dermatologic care, and clinical trials in some areas.

## 3 Which Acne Patients Can Benefit From Dermocosmetics

### 3.1 Milder Forms of Acne

**FIGURE 2:**
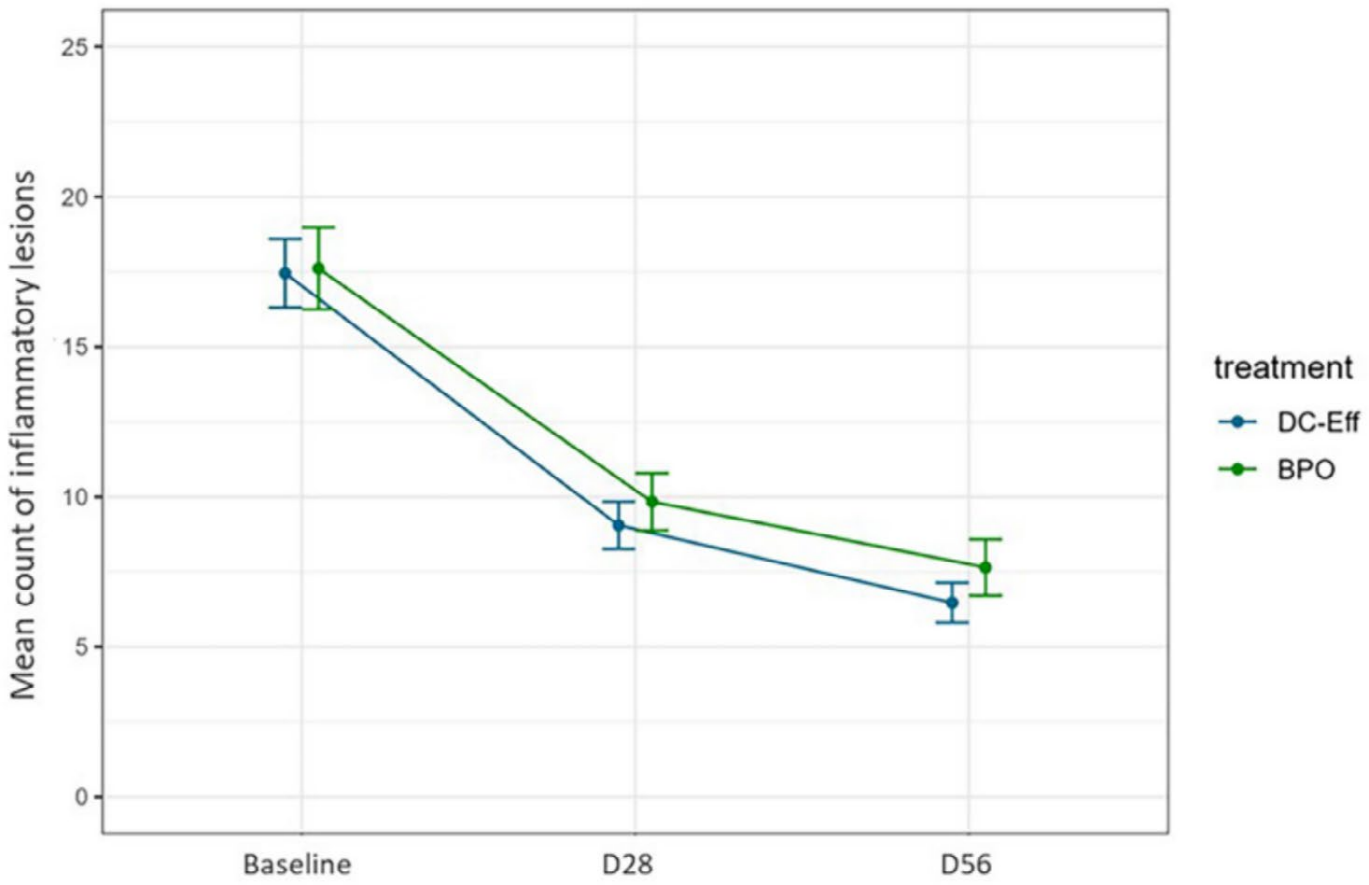
Reductions in lesion counts with salicylic acid‐based moisturizer and BPO; (*p* < 0.001 for both groups). From Dal Belo et al. [18].

For milder forms of acne (few inflammatory and non‐inflammatory lesions with or without excess facial oiliness), it is reasonable to consider a trial of dermocosmetics alone [11]. In a recent detailed review, Thiboutot et al. recently reported that many studies have evaluated dermocosmetic use alone in milder forms of acne [11]. Two illustrative examples include Bissonnette et al. and Dal Belo et al. [17, 18] Bissonnette et al. conducted a 12‐week randomized controlled trial (RCT) compared 0.3% LHA formulation and 5% benzoyl peroxide in 80 patients with mild–moderate acne [17]. Acne lesions were decreased (*p* < 0.05) in both groups to a comparable degree [17]. Both products were also well tolerated, with a trend toward better tolerability with LHA than BPO [17]. In a second RCT, Dal Belo et al. evaluated a multitargeted salicylic acid‐based (salicylic acid, LHA, niacinamide, piroctone olamine, zinc, *Vitroscella filiformis*, and 2‐oleamideo‐1,3‐octadecanediol) moisturizer and BPO 5% in 150 adult patients (18–40 years old) [18]. In this 8‐week parallel‐group study of adult acne, acne lesion counts were significantly decreased (*p* < 0.001) with both groups (Figure 2), with a trend to greater reductions in the salicylic acid‐based moisturizer group compared to BPO [18]. In addition, the salicylic acid‐based moisturizer was better tolerated and patients reported that their skin was smoother [18].

Overall, these studies indicate that dermocosmetics can be an option for milder acne, either alone or as a first step before prescription treatment.

### 3.2 Oily Skin

Many patients in Latin America report having oily skin, and excess sebum production is an important pathophysiologic pathway in acne [5]. Further, Kaminsky et al. report that oily skin in Latin America is a risk factor associated with more severe forms of acne [5]. In a 2022 study, Do et al. found that human lipids play a role in driving inflammation [19]. These researchers found that TREM2 macrophages in the skin affect lipid metabolism and increase inflammation near hair follicles expressing a squalene catalytic enzyme, which leads to alterations in the macrophages that make them unable to successfully kill *C acnes* [19]. In the same study by Kaminsky, 43% of included patients were Mestizos (skin types II–IV) and there was a 70.8% rate of hyperseborrhea and a not insignificant proportion of patients had signs of hyperandrogenism (hirsutism, alopecia, and acanthosis nigricans). In the setting of oily skin, which may be more intense in adult females with mild acne, it is important that the skin care regimen matches the skin type [5]. Cleansers should be rinsable products that have no residual moisturizer, while moisturizers should include anti‐acne ingredients [20]. Minimizing surface oiliness can have an additive effect with medical treatments [20].

Taken together, these data suggest that dermocosmetic regimens adapted to oily, acne‐prone skin can improve sebum control and support medical therapy.

### 3.3 Case Study

**FIGURE 3:**
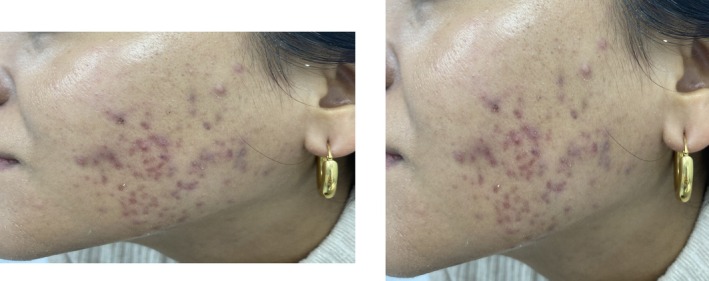
Case patient photo. Courtesy of Dr. Adriana Gamarra.

A 33‐year‐old female with Fitzpatrick skin phototype IV and mild acne (Figure 3) was managed with minocycline 100 mg plus spironolactone 100 mg plus a topical dermocosmetic regimen consisting of an oily skin cleanser; a serum containing salicylic acid, glycolic acid, niacinamide, and tranexamic acid; and sunscreen. The patient also received dietary counseling to include omega 3 fatty acids.

## 4 Targeting PIH as a Complication

Rocha et al. have reported that most Latin American patients with acne have an increased risk of acne‐induced hyperpigmentation, with darker skin phototypes that may be in part due to migratory factors and miscegenation [1]. Dermocosmetics with bleaching action can be associated with acne treatment; ingredients of interest have anti‐inflammatory and anti‐pigmenting actions and may include niacinamide, licochalcone, retinoids, thiamidol, and alpha arbutine [1].

Giavina‐Bianchi et al. assessed 2459 acne patients in Brazil and found PIH in more than 53% of patients [21]. Studies have reported that PIH can be very long lasting and often has an impact on quality of life that is more dramatic than that of the active acne itself [22, 23]. Benzaquen et al. reported that use of a dermocosmetic over 8 weeks reduced PIH associated with acne in patients with Fitzpatrick skin phototypes IV–VI [24]. The average number of PIH marked was reduced from 35.6 at baseline to 28.6 at day 56 (*p* < 0.001) [24].

**FIGURE 4:**
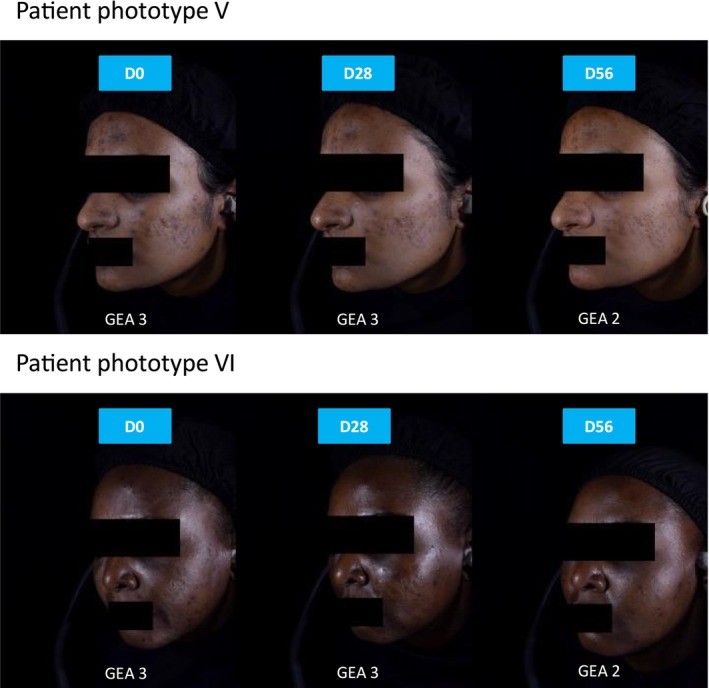
Clinical representations of reduction in PIH marks.

Overall, the available evidence shows that dermocosmetics with anti‐inflammatory and depigmenting ingredients may help reduce acne‐related PIH, especially in darker phototypes (Figure 4).

## 5 Adjunctive to Complement Efficacy

A number of controlled studies have been published evaluating use of dermocosmetics as adjunctive treatments, which evaluated impact on efficacy and tolerability [25, 26, 27, 28, 29, 30, 31, 32, 33, 34, 35, 36, 37, 38, 39, 40]. These studies show that dermocosmetics not only decreased acne lesions and improved global assessments but also achieved improvements in quality of life, patient satisfaction, treatment adherence, and skin hydration over prescription therapy alone when these parameters were evaluated [11].

Collectively, these trials support the use of dermocosmetics as adjuncts to prescription acne treatments to improve lesion outcomes and patient‐reported measures such as satisfaction and adherence.

## 6 Adjunct to Mitigate Side Effects

In addition to improving efficacy and adherence to prescription medications, dermocosmetic‐containing products can also improve tolerability and safety [11]. This may be most useful in patients with moderate to severe acne, those who are being treated with potentially irritating medications (topical retinoids, BPO, isotretinoin), and those who have reduced adherence due to poor tolerability [25, 26, 29, 30, 34, 38, 39, 40].

Taken together, these findings indicate that dermocosmetic regimens can reduce local side effects of acne treatments and improve overall tolerability.

## 7 Illustrative Case Study

**FIGURE 5:**
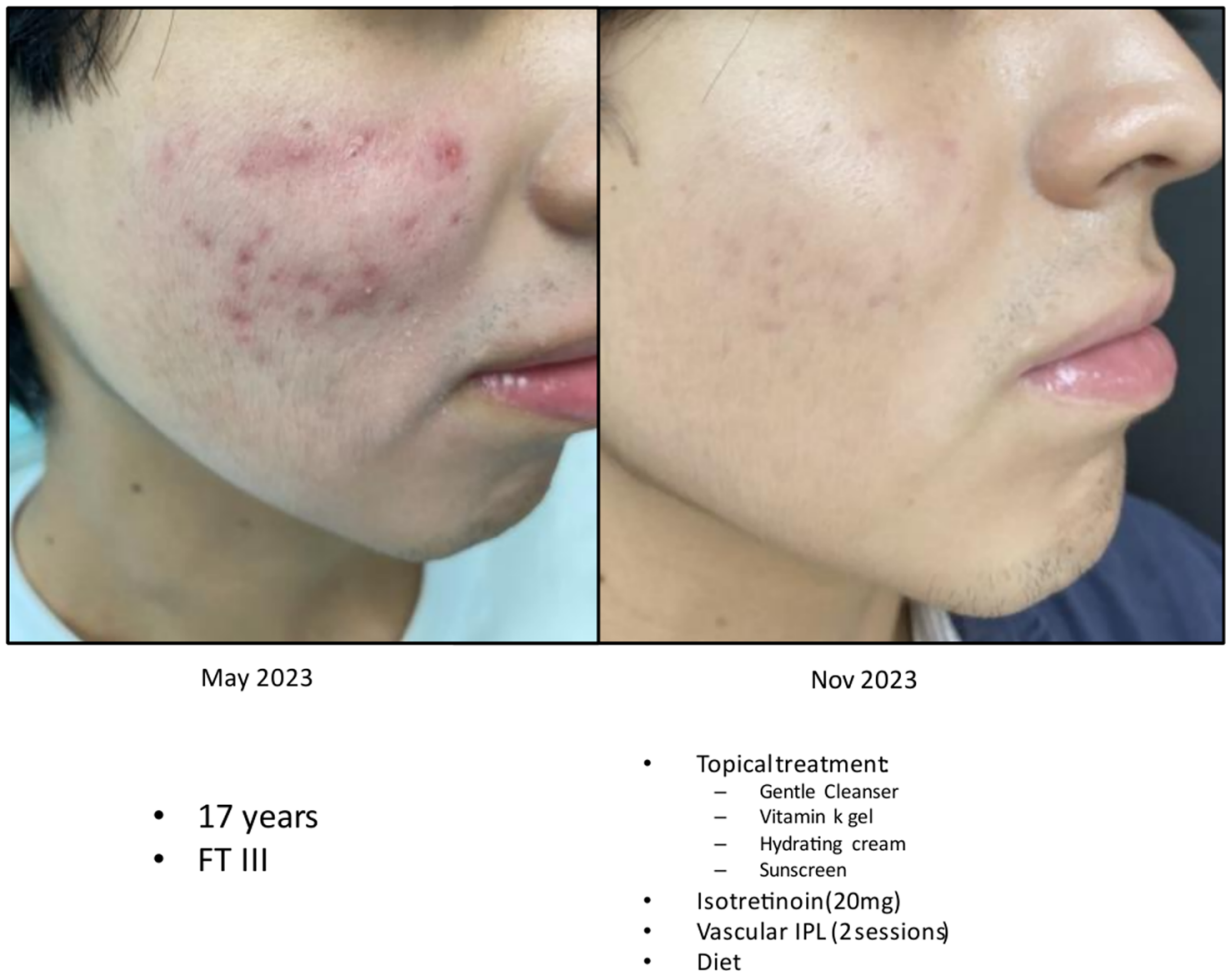
Clinical representation of dermocosmetic use in combination with isotretinoin. Photo courtesy of Dr. Adriana Gamarra.

A 17‐year old male with Fitzpatrick type III skin underwent isotretinoin treatment accompanied by a daily regimen of a gentle cleanser, a vitamin K gel, and a moisturizer plus sunscreen. Figure 5 shows the improvement in acne from May 2023 to November 2023.

## 8 Maintenance

Dreno et al. studied a salicylic acid‐based moisturizer versus a traditional moisturizer as maintenance in an 18 week RCT and reported reduced relapse of non‐inflammatory lesions in the dermocosmetic group compared to those managed with the traditional moisturizer [41]. Later, Khammari et al. conducted a large 12‐week RCT of a salicylic acid‐based moisturizer versus its vehicle as maintenance after treatment for mild to moderate acne in 100 patients aged 15–30 years [25]. These researchers reported that acne continued to improve in the dermocosmetic group in contrast to relapsing in the vehicle group [25]. Kulthanan et al. assessed the impact of a dermocosmetic containing licochalcone A, decanediol, L carnitine, and salicylic acid compared with its vehicle in adult patients (*n* = 50, 18 years or older) with mild to moderate acne who had achieved at least a 50% improvement with treatment over a 12 week period [42]. Similar to the Khammari study, acne lesions continued to improve with dermocosmetic yet increased with vehicle [42]. In an open‐label study, Queille‐Roussel studied a triple acid complex serum in adult female acne (*n* = 30) and reported that acne lesions continued to decrease after treatment and there was no clinical relapse [43].

Overall, the available data suggest that dermocosmetics may help maintain acne improvement and reduce the risk of relapse after active treatment.

## 9 Illustrative Case Study

**FIGURE 6:**
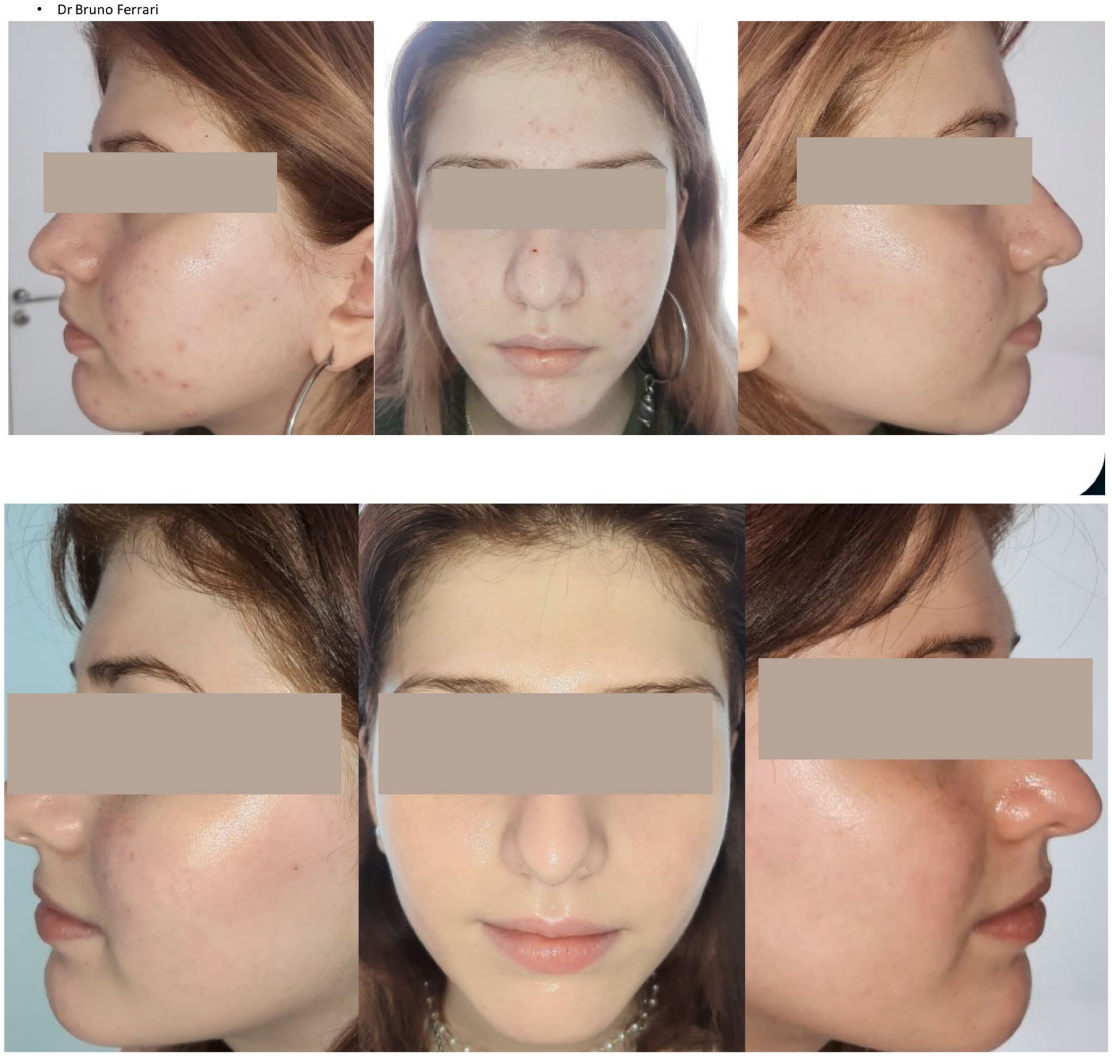
Clinical representation of dermocosmetics used as maintenance therapy. Photo and case courtesy of Dr. Bruno Ferrari.

A 12 year old female with mild acne with adapalene/BPO with adjunctive moisturizers (Figure 6). Once clearance was obtained, the patient was continued on a dermocosmetic regimen (cleanser, serum, and moisturizer) with the active ingredients salicylic acid, LHA, niacinamide, glycolic acid, and zinc.

## 10 Conclusions

The positive impact of dermocosmetics in acne management is increasingly reported in the medical literature and should be recognized by healthcare professionals managing patients with acne. In particular, dermocosmetics may be considered (1) as monotherapy for milder forms of acne or an alternative to medical treatment if patients wish to initiate therapy in a conservative manner or have had poor past experiences, (2) as monotherapy for maintenance after medical treatment, (3) as adjuncts to complement the mode of action and efficacy of medical treatments, and (4) as adjuncts to mitigate irritation. It is important to improve awareness and information with both professional and lay populations about acne, its sequelae, and manifestations within Latin America. Whenever possible, it is also desirable to minimize barriers to access to good acne management strategies and treatments.

## Author Contributions

All authors participated in writing of the manuscript or critical review of important intellectual content and gave final approval of the final manuscript.

## Funding

Funding for editorial support was provided by La Roche‐Posay Laboratoire Dermatologique, L’Oreal Dermatological Beauty Division.

## Ethics Statement

The authors have nothing to report.

## Consent

All patients who participated in the cases presented in this publication provided written informed consent.

## Conflicts of Interest

Dr Troielli served as a consultant/received honoraria from Beiersdorf, Galderma, La Roche Posay, and L'Oreal. Dr Moreno, Dr Gamarra, and Dr Cortes have served as consultants for L'Oreal. Dr Cardenas has served as a consultant for La Roche Posay, Galderma, and Isdin. Dr Kerob is an employee of L'Oreal. Pr Dreno has served as consultant/received honoraria from Bristol Meyers Squibb, Almirall, Galderma, La Roche Posay, Pierre Fabre, and Bioderma.

## Data Availability

Data sharing not applicable to this article as no datasets were generated or analyzed during the current study.
